# Application of response surface methodology for modeling adsorption of janus green and safranin-O on magnetic nanocomposite from aqueous solutions

**DOI:** 10.1038/s41598-025-02644-1

**Published:** 2025-08-25

**Authors:** Irfan Ahmad, Dilshad A. H. Alhadrawi, Bhavesh Kanabar, T. Ramachandran, Haider Radhi Saud, Aman Shankhyan, A. Karthikeyan, Dhirendra Nath Thatoi

**Affiliations:** 1 Department of Clinical Laboratory Sciences, College of Applied Medical Sciences, King Khalid University, Abha, Saudi Arabia; 2 Department of Computer Techniques Engineering, College of Technical Engineering, The Islamic University, Najaf, Iraq; 3 Department of Computer Techniques Engineering, College of Technical Engineering, The Islamic University of Al Diwaniyah, Al Diwaniyah, Iraq; 4 Department of Computer Techniques Engineering, College of Technical Engineering, The Islamic University of Babylon, Babylon, Iraq; 5 Department of Mechanical Engineering, Faculty of Engineering and Technology, Marwadi University Research Center, Marwadi University, Rajkot, Gujarat 360003 India; 6 Department of Mechanical Engineering, School of Engineering and Technology, JAIN (Deemed to be University), Bangalore, Karnataka India; 7 College of Health and Medical Technology, National University of Science and Technology, Nasiriyah, Dhi Qar 64001 Iraq; 8 Centre for Research Impact and Outcome, Chitkara University Institute of Engineering and Technology, Chitkara University, Rajpura, Punjab 140401 India; 9 Department of Mechanical Engineering, Sathyabama Institute of Science and Technology, Chennai, Tamil Nadu India; 10 Department of Mechanical Engineering, Siksha ‘O’ Anusandhan (Deemed to be University), Bhubaneswar, Odisha 751030 India

**Keywords:** Response surface methodology, Janus green, Safranin-O, Removal, Environmental sciences, Chemistry

## Abstract

The presence of dyes in water bodies has become a significant concern in recent years. In this study, a magnetic nanocomposite synthesized from activated carbon (AC) and Fe_3_O_4_ nanoparticles (AC/FeO) was employed as an adsorbent to remove janus green (JG) and safranin-O (SO) dyes. The AC/FeO nanocomposite had particle sizes smaller than 100 nm. The specific surface area, pore size, and pore volume average of the AC/FeO nanocomposite were 329.56 m^2^ g^−1^, 3.67 nm, and 0.289 cm^3^ g^−1^, respectively. The pH_pzc_ of AC/FeO magnetic nanocomposite was found to be 5.3. Optimal conditions for removal were determined to be a dye concentration of 20 mg L^−1^, a sonication time of 18 min, a nanocomposite dosage of 0.023 g, and a pH of 7. The AC/FeO nanocomposite exhibited excellent adsorption performance, achieving removal efficiencies of 95.79% and 97.60% for JG and SO, respectively. Moreover, the magnetic nanocomposite demonstrated good reusability, making it a promising candidate for removing JG and SO dyes from aqueous solutions. The AC/FeO nanocomposite was used to remove JG and SO from different environmental water samples, and the removal efficiency was obtained in the range of 87.65–94.81%. Overall, this study highlights the potential of the AC/FeO magnetic adsorbent, as an effective adsorbent for removing JG and SO dyes from water, which could have significant implications for water treatment and environmental protection.

**Supplementary Information:**

The online version contains supplementary material available at 10.1038/s41598-025-02644-1.

## Introduction

The rapid growth of the global population and overexploitation of limited water resources and their contamination have raised serious concerns about an impending water crisis in the coming years due to various biological, agricultural, and industrial activities^1,2^. According to projections, the demand for water, energy, and food is expected to rise significantly by 2050 compared to current levels^3^. Consequently, the depletion of water resources and the decline in water quality have led to the widespread implementation of wastewater reclamation and treatment programs. In this respect, ensuring water safety is critical to reusing treated water^4^.

Dyes and pigments are vital in various industries, including textiles, paper production, food processing, and cosmetics. However, the discharge of these substances into aquatic environments has emerged as a major environmental issue^5^. Dye-laden wastewater, often released from industrial processes, can adversely impact aquatic ecosystems and pose risks to human health. Such wastewater contains complex and resistant compounds that are typically difficult to biodegrade, allowing them to persist in the environment. The present study investigates the presence of dyes in aquatic systems and their harmful effects^6,7^.

Janus green (JG) is a chemical dye from the basic dye family, widely used in cellular biology and microscopic studies. This dye holds a unique position in biological research considering its ability to identify and differentiate specific cellular organelles, such as mitochondria, particularly in live or freshly prepared samples^8^. As a biological marker, JG provides valuable insights into cellular metabolic activity by changing its color in the presence of oxygen and oxidative variations. Nevertheless, at high concentrations, JG can be toxic to living cells and organelles. This toxicity may disrupt mitochondrial function and lead to cell death^9^. Besides, like many chemical dyes, improper disposal of JG can result in water and soil contamination, posing a significant threat to natural ecosystems. Direct contact with JG may cause irritation to the skin, eyes, and respiratory system. Accidental inhalation or ingestion of this compound may also result in toxicity issues^10^.

Safranin-O (SO) is a chemical dye from the basic dye family, widely utilized in biology, microscopy, and various industries. Regarding its strong staining properties, SO is commonly employed as a counterstain in techniques such as Gram staining and for identifying cellular structures in biological samples. This dye is particularly useful for staining cell nuclei and cell walls in light microscopy, enhancing the differentiation of structures under the microscope^11,12^. Additionally, SO is applied in histology to identify cartilage and other body tissues. In industrial applications, it is used for dyeing fibers and leather. However, direct contact with SO may cause skin and eye irritation, leading to redness^13^. Accidental ingestion or inhalation of this dye can result in gastrointestinal and respiratory discomfort. Despite its numerous applications across various fields, the improper release of JG and SO dyes into the environment can have detrimental effects on aquatic ecosystems, posing toxicity risks to aquatic organisms. Conventional water and wastewater treatment processes cannot effectively degrade and remove JG and SO dyes. Accordingly, several methods, such as photocatalysis, ozonation, electrocoagulation, and adsorption, have been employed to eliminate these compounds^14–19^.

Among the mentioned techniques, adsorption has garnered significant attention due to its high efficiency, operational flexibility, and cost-effectiveness in removing pollutants^20–22^. Carbon-based adsorbents, particularly activated carbon (AC), have been widely used for dye removal from surface waters owing to their ease of use, large surface area, and high efficiency^23,24^. The AC refers to a group of porous carbon materials with a high internal surface area, significant internal area, surface reactivation capability, high adsorption capacity, porous structure, and relatively low cost compared to inorganic adsorbents^25,26^.

However, the primary challenge in using powdered AC or nano-sized adsorbents lies in the difficulty of separating them from the solution due to their small particle size, which can lead to dispersion and secondary pollution^27^. Magnetizing such adsorbents has been proposed as an effective solution to address these issues. In this context, the use of magnetic nanoparticles to produce adsorbents with a high active surface area has proven highly effective. The explanation is that their porous structure and large surface area enhance adsorption efficiency for pollutant removal^28^. Furthermore, magnetic nanoparticles offer the advantage of easy separation from aqueous solutions. Because of their low toxicity and cost-effectiveness, magnetic nanoparticles are economically viable for removing various pollutants^29^. In the present study, an AC/FeO composite was synthesized and used to remove JG and SO dyes from aqueous solutions.

Banadaki et al. (2024) investigated the removal of thorium (IV) using magnetic AC derived from date palm fibers (AC/FeO). The results demonstrated that this process could effectively remove thorium (IV) within a short period. Statistical analysis revealed that the parameters of concentration, pH, adsorbent dosage, and contact time significantly influenced the removal efficiency of thorium (IV). In this research, a removal efficiency of 96.60% was achieved under optimal conditions (i.e., contact time of 30 min, adsorbent dosage of 3.12 g, pH of 4, and an initial concentration of 11 mg L^−1^)^30^.

D′Cruz et al. (2020) examined the efficiency of Fe_3_O_4_ magnetic AC nanocomposite (AC/FeO) in removing promazine from aqueous solutions. They showed that the initial concentration, adsorbent dosage, solution pH, and contact time significantly affected the removal efficiency. The highest removal rate of promazine (99.97%) was achieved at a pH of 8.5 and a contact time of 6 min. The nanocomposite exhibited minimal reduction in efficiency after five adsorption cycles, demonstrating its potential as a robust platform for removing pollutants from wastewater^31^.

In another study, Jafari et al. (2017) evaluated the performance of AC/FeO adsorbent in removing tetracycline (TC) from aqueous environments. It was observed that parameters such as pH, initial concentration, and adsorbent dosage significantly affected the removal efficiency of TC. Under optimal conditions, a removal efficiency of 99.80% was achieved. Given the promising results, the AC/FeO adsorbent proved to be a reliable method for removing TC from industrial wastewater^32^.

In cases where the interaction between variables influences the outcome of a process, statistical methods can be employed as a suitable approach for experimental design. These methods help reduce material consumption, experimental costs, and the time required for conducting experiments^33^. When a combination of several independent variables and their interactions affects the desired response, the response surface methodology (RSM) serves as an effective tool for process optimization. RSM employs an experimental design to fit a model using the least squares technique^34^. The adequacy of the proposed model is then evaluated through diagnostic tests provided by analysis of variance (ANOVA). RSM plots can be used to assess the response surfaces and determine the optimal values. In several treatment processes, RSM has been routinely applied to evaluate results and operational efficiency^35,36^.

In the present study, the efficiency of JG and SO dye removal as emerging pollutants was investigated using the performance of the AC/FeO magnetic nanocomposite. The removal was evaluated based on CCD, considering key variables such as nanocomposite dosage, dye concentration, pH, and sonication time.

## Experimental

### Materials and equipment

The materials used in this research included iron(III) chloride hexahydrate (FeCl_3_·6H_2_O, ≥ 99%), safranin-O (C_20_H_19_ClN, ≥ 85%), sodium hydroxide (NaOH, ≥ 97%), janus green (C_30_H_31_ClN_6_, ≥ 65%), hydrochloric acid (HCl, ≥ 37%), and iron(II) chloride tetrahydrate (FeCl_2_·4H_2_O, ≥ 99%). All chemicals were procured from Merck and Sigma-Aldrich and used as received without further purification. The pH of the aqueous solutions was adjusted using NaOH (0.1 M) and HCl (0.1 M) and measured with a pH meter (Orion 5-star, Thermo Scientific, USA). Double-distilled water was employed during the synthesis and washing processes. Dye concentrations were determined using a UV/Vis spectrophotometer (Lasany Li-2800, London). We used an ultrasonic bath (KQ-300VDE, China) to ensure optimal dispersion of the solution’s adsorbent and facilitate adsorption and desorption. Fourier-transform infrared spectroscopy (FT-IR, Bruker Victor 22, Germany) was employed to identify functional groups. The crystalline phases and morphology of the synthesized adsorbents were characterized by X-ray diffraction (XRD, Stoe StadiP, Germany) and scanning electron microscope (SEM, Hitachi S-4800, Japan). The Brunauer–Emmett–Teller (BET) surface areas were measured using BELSORP Mini II (BEL Japan). Vibrating-sample magnetometry (VSM, Lake Shore 7400, USA) was used to assess the magnetic properties of Fe_2_O_4_ nanoparticles and the synthesized composite.

### Synthesis of AC/FeO nanocomposite

The AC/FeO nanocomposite was synthesized using the chemical precipitation method. For this purpose, walnut shells were washed twice with distilled water to remove surface impurities. Next, they were dried in an oven at 400 °C for 2 h and subsequently ground into a fine powder. The resulting powder was activated by immersion in a 1 M NaOH solution. The AC was then separated from the solution and placed in a furnace at 700 °C for 2 h. After cooling to room temperature, the powder was washed several times with distilled water until its pH reached 7, ensuring neutrality. In the next step, 0.5 g of the AC was dispersed in 100 mL of deionized water under ultrasonic waves for 15 min. Subsequently, 2.7 g of FeCl_3_ and 0.99 g of FeCl_2_ (at a molar ratio of 2:1) were added to the mixture under vigorous stirring. NaOH was then added dropwise as an oxidizing agent until the pH of the solution reached 11. After 60 min, the resulting precipitate was collected using an external magnet, washed several times with deionized water, and finally dried at 80 °C for 24 h^37^. The morphological properties of the AC/FeO nanocomposite were characterized using VSM, BET, FT-IR, XRD, and SEM analyses.

### Removal experiments

This study was conducted in batch mode using JG and SO dyes. The investigation focused on four key variables: sonication time, pH, dye concentration, and the amount of magnetic nanocomposite. These variables were adjusted within specific ranges to determine the optimal conditions for each parameter. Stock dye solutions with a concentration of 1000 mg L^−1^ were prepared for the experiments. The tests were carried out in 250 mL Erlenmeyer flasks containing 100 mL of dye solution at different concentrations. The pH of the solutions was adjusted using HCl and NaOH according to the experimental design table, and the required amount of adsorbent was weighed and added to the solution. Samples were collected at intervals of 5, 10, 15, 20, and 25 min. The adsorbent was then separated from the solution using a magnetic separator, and the removal efficiency of JG and SO dyes was determined using a UV/Vis spectrophotometer. The removal efficiency was calculated using Eq. (1).

1 $$\%Removal = \frac{{C}_{0}-{C}_{e}}{{C}_{0}}\times 100$$

where *C*_*0*_ and *C*_*e*_ denote the initial and final concentrations of the solution, respectively^38^.

### Experimental design

RSM is among the chemometric methods used to optimize variables affecting the process and examine the response of the interactions. This method optimizes a response (output variable) affected by several independent variables by precisely designed tests^39^. This response can be used to obtain the most reliable response with the minimum number of tests. The advantages of using RSM include investigating the interactions of factors affecting the process and reducing the number of tests, saving the consumption of raw materials, and reducing the time the tester spends^40^. The CCD is one of the most well-known types of RSM designs. Initial concentration, ultrasound radiation time, pH, and composite content were selected as independent parameters, and the removal percentage as the dependent (independent) parameters. The design was prepared using the CCD method with Design Expert 12 software. Table 1 lists the variables and their selected ranges in the CCD method, which were defined as model inputs.

**Table 1:** The design of CCD.

Variables	Symbol	Unit	Levels					Step change value
			− 2	− 1	0	+ 1	+ 2	
AC/FeO amount	A	g	0.010	0.015	0.020	0.025	0.030	0.005
pH	B	–	4	5	6	7	8	1
Concentration	C	mg L^−1^	10	20	30	40	50	10
Sonication time	D	min	5	10	15	20	25	5

The number of tests required for the f-factor in the design was calculated using Eq. (2).

2 $$\text{N}= {2}^{f}+ 2f + C$$

where *f* and *C* are the numbers of variables and central points, respectively^41^. Based on Eq. (2), 30 tests are necessary for this design. Simple or multiple linear regression is among the most widely used statistical methods for data analysis in various sciences. This method examines the relationships between variables and whether or not one variable can influence another. More precisely, a regression equation can be written based on the knowledge of one or more independent variables to predict the dependent variable’s values. Based on the tests and data recording, the relationship between the process response and independent parameters is expressed as a quadratic model (Eq. 3).

3 $$\text{Y}= {a}_{0}+ \sum_{i=1}^{n}{a}_{i}{X}_{i} + {\sum }_{i=1}^{n}{a}_{ii}{X}_{i}^{2} + {\sum }_{i=1}^{n-1}{\sum }_{i=2}^{n}{a}_{ij}{X}_{i}{X}_{j} + e$$

where *Y* represents the response, *i* and *j* are the linear and quadratic coefficients, *a*_*0*_ is the constant term, *a*_*i*_ denotes the linear coefficient, *a*_*ii*_ represents the interaction effect coefficient, *a*_*ij*_ is the quadratic coefficient, and *e* is the random error^42^. The accuracy of the fitted model was evaluated using the ANOVA results of the obtained laboratory data. The results obtained from the model with the laboratory data were verified by conducting three additional tests under optimal conditions, followed by comparing the laboratory results with the values predicted by the model. Two- and three-dimensional plots were drawn based on the influence of independent factors to depict the removal rate of heavy metals.

## Results and discussions

### Characterization studies

The FT-IR analysis was conducted to investigate and identify the functional groups present in the structures of AC/FeO, AC, and Fe_3_O_4_, with the results shown in Fig. S1. The first and second spectra exhibit a strong absorption peak at 3676 cm⁻^1^, corresponding to the free H–O bonds in their structures. Additionally, other absorption peaks at 1523 cm⁻^1^ and 1647 cm⁻^1^ are observed, which are attributed to the C=C bonds of alkenes and aromatic compounds present in the AC structure, respectively. Furthermore, a prominent peak at 1743 cm⁻^1^ indicates the presence of CO bonds in the AC derived from walnut shells^43^. After incorporating iron oxide into the AC structure and forming the AC/FeO composite, in addition to the absorption maxima observed in the AC structure (with slight shifts and changes in the spectrum), a peak at 9675 cm⁻^1^ appears, corresponding to free OH groups. Also, a peak at 9029 cm⁻^1^ corresponding to the C=C bonds of alkenes in the AC structure is noticed. Moreover, a peak at 569 cm⁻^1^ is associated with Fe–O bonds, indicating the presence of Fe_3_O_4_. Another absorption peak at 9455 cm⁻^1^ is attributed to the OH bonds present in the structure.

The XRD analysis was conducted for Fe_3_O_4_, AC, and AC/FeO within the range of 10° to 80°, and the results are shown in Fig. S2. According to this figure, peaks with varying intensities appear in the composite structure, indicating the presence of both crystalline and semi-crystalline phases. Based on these results, it can be concluded that the composite has a crystalline structure. In the X-ray diffraction pattern of AC/FeO, peaks at diffraction angles (2θ) of 20.30° and 43.60° correspond to the (002) and (001) crystalline phases, respectively, associated with the graphite structure in AC. This result has also been confirmed by previous studies^44^. Other peaks at 2θ values of 29.65° (220), 40.15° (222), and 64.90° (440) can be attributed to the presence of AC in the structure^45^. Moreover, in the AC/FeO magnetic composite, additional peaks at 2θ values of 30.40°, 35.85°, 43.50°, 57.45°, and 63.25° are observed, corresponding to the (220), (311), (400), (422), (511), and (440) crystalline phases of Fe_3_O_4_ nanoparticles. These findings confirm that the Fe_3_O_4_ nanoparticles formed within the AC possess an inverse spinel cubic structure^44^. Furthermore, using the Debye–Scherrer equation, the crystal size of the AC/FeO composite was calculated to be approximately 17 nm.

The magnetic properties of Fe_3_O_4_ nanoparticles and the synthesized magnetic composite were analyzed using vibrating-sample magnetometry (VSM). The results (Fig. S3) indicate that the samples’ remanence and coercivity are zero, as the hysteresis loops are perfectly symmetrical and pass through the origin. These findings confirm that the nanoparticles and the composite exhibit superparamagnetic behavior. Therefore, magnetic separation and their reusability are feasible^46^. Moreover, the results showed that Fe_3_O_4_ nanoparticles have a higher saturation magnetization (84.36 emu g^−1^) than the AC/FeO composite (30.93 emu g^−1^). The reduction in the saturation magnetization of the composite can be attributed to factors such as the extensive coating of Fe_2_O_4_ with a non-magnetic matrix (AC)^47^.

The morphology and surface characteristics of AC derived from walnut shells and the AC/FeO composite were examined using SEM analysis. As shown in Fig. S4, the AC produced from walnut shells exhibits a highly porous and heterogeneous surface, which can play a significant role in adsorption processes. In Fig. S4, after the deposition of Fe_3_O_4_ nanoparticles onto the AC surface, spherical particles are visible on the surface. These particles likely result from the formation of Fe_2_O_4_ nanoparticles on the AC, leading to surface modifications and increased surface roughness.

BET analysis was used to determine the specific surface area, pore size, and volume of AC/FeO nanocomposite (Fig. S5). The results showed that AC/FeO nanocomposite had a specific surface area of 329.56 m^2^ g^−1^. Also, the pore size and pore volume average were obtained as 3.67 nm and 0.289 cm^3^ g^−1^, respectively. These results indicate that the synthesized adsorbent has a highly porous structure, facilitating molecular diffusion and providing sufficient space for dye molecules’ adsorption from water. According to the International Union of Pure and Applied Chemistry (IUPAC) classification, the structure of a porous medium, based on the average pore size, can contain pores smaller than 2 nm called micropores, pores between 2 and 50 nm called mesopores, and pores larger than 50 nm called macropores^48^.

### Determining the point of zero charge (pHpzc)

**Fig. 1:**
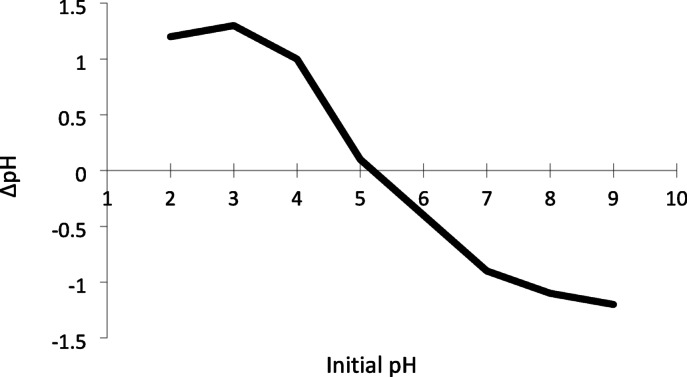
The pH_pzc_ curve of AC/FeO nanocomposite.

The pH_pzc_ of the AC/FeO nanocomposite was determined using the Salt Addition method, which is a common approach for measuring pH_pzc_. For this purpose, KCl solutions with a fixed concentration (0.01 M) were prepared as the background electrolyte. Eight KCl solutions (50 mL) with different initial pH values (2–9) were adjusted by adding precise amounts of HCl (0.01 M) or NaOH (0.01 M). A pH meter was used to measure and adjust the pH values. Subsequently, a specific amount of the AC/FeO composite (0.1 g) was added to each KCl solution. The mixtures were stirred continuously at a constant temperature for 24 h to reach equilibrium. After equilibrium was achieved, the final pH of each solution was measured using the pH meter. The change in pH (ΔpH) was calculated as the difference between the initial and final pH values (ΔpH = pH_fiinal_–pH_initial_). A plot of versus was then created. The point where ΔpH = 0 was identified as the pH_pzc_. According to Fig. 1, the pH_pzc_ of the AC/FeO nanocomposite was determined to be 5.3.

### RSM based on CCD

The research design using the CCD method, the levels of independent variables, and the laboratory results (the response variable) are represented in Table 2.

**Table 2:** The results of DOE.

Variables					%Removal of JG		%Removal of SO	
Run	A (g)	B	C (mg L^−1^)	D (min)	Predicted	Experimental	Predicted	Experimental
1	0	− 2	0	0	23.40	23.88	39.04	39.51
2	1	1	1	1	72.74	72.67	77.82	77.52
3	1	− 1	− 1	− 1	59.82	60.20	63.91	63.36
4	0	0	− 2	0	82.35	83.46	84.86	84.93
5	− 1	− 1	1	1	34.94	35.08	41.47	41.50
6	1	1	− 1	− 1	74.05	72.97	79.97	79.83
7	0	0	0	0	81.57	80.36	86.33	85.99
8	0	0	0	2	77.90	78.59	75.71	75.88
9	0	0	0	0	81.57	83.28	86.33	86.71
10	0	0	2	0	45.00	45.17	43.73	44.25
11	0	0	0	0	81.57	82.71	86.33	86.18
12	− 1	1	− 1	− 1	51.09	50.97	65.88	65.89
13	1	− 1	− 1	1	70.70	69.78	75.42	75.74
14	− 1	− 1	− 1	− 1	37.10	36.22	57.41	57.60
15	1	1	− 1	1	91.40	90.86	92.42	92.19
16	− 1	1	− 1	1	69.98	69.82	71.44	71.61
17	− 1	1	1	1	53.22	52.50	51.56	51.63
18	1	1	1	− 1	51.28	51.11	59.40	59.42
19	0	0	0	− 2	44.03	44.62	52.67	53.09
20	− 1	− 1	− 1	1	49.51	49.34	62.02	61.52
21	0	2	0	0	55.90	56.70	65.19	65.31
22	0	0	0	0	81.57	79.95	86.33	86.41
23	0	0	0	0	81.57	81.48	86.33	87.07
24	− 1	− 1	1	− 1	18.42	18.62	30.88	30.63
25	− 1	1	1	− 1	30.22	30.20	40.03	39.59
26	1	− 1	1	− 1	39.24	38.46	42.66	42.38
27	2	0	0	0	71.32	72.38	78.90	79.43
28	− 2	0	0	0	29.08	29.30	46.14	46.20
29	1	− 1	1	1	54.23	54.01	60.15	59.66
30	0	0	0	0	81.57	81.61	86.33	85.63

Based on the results, an experimental relationship between the response (removal percentage) and independent variables was extracted using the actual values. Equations (4) and (5) express this quadratic polynomial equation.

4 $$\begin{aligned} \% {\text{Removal of JG}} & = + 82.33 \, + 10.43{\text{A }}7.91{\text{B}} - 9.21{\text{C }} + 8.51{\text{D}} + 0.24{\text{AB}} \\ & \quad - 0.66{\text{AC}} - 0.32{\text{AD}} - 0.73{\text{BC}} + 1.43{\text{BD}} + 0.96{\text{CD}} \\ & \quad - 7.64{\text{A}}^{2} - 10.15{\text{B}}^{2} - 4.27{\text{C}}^{{2}} - 5.07D^{2} \\ \end{aligned}$$

5 $$\begin{aligned} \% {\text{Removal of SO}} & = + {92}.{35} + {6}.{\text{49A}} + {8}.{\text{44B}} - {8}.{6}0{\text{C}} + {4}.{\text{27D}} \\ & \quad + {1}.{\text{21AB}} - 0.{\text{43AC}} + 0.{\text{47AD}} + 0.{\text{34BC }} \\ & \quad - 0.{\text{75BD}} + 0.{\text{56CD}} - {6}.{\text{81A}}^{2} - {7}.{\text{71B}}^{2} - {5}.{\text{83C}}^{2} - {5}.{\text{57D}}^{2} \\ \end{aligned}$$

where *A*, *B*, *C*, and *D* correspond to the AC/FeO amount, pH, concentration, and sonication time, respectively. The accuracy of the fitted model was verified using ANOVA results. According to Table 3, an error level of < 0.05, a *p*-value of < 0.05 (0.0001 for both models), and high F-values of the model (360.24 and 188.24 for JG and SO, respectively) show the significance of the model. Based on the *p*-value definition, there is a probability of only 0.01% that a high F-value results from noise. In other words, this high value arises from the high influence of controllable factors (signal) on uncontrollable ones (noise) in the model. The coefficient of determination (R^2^), as a criterion representing the total changes in the model-predicted response, specifies the sum of squares resulting from the total regression to the sum of squares. An R^2^ value close to 1 is desirable, and a satisfactory agreement is necessary with the adjusted R^2^ (Adj-R^2^). In this study, R^2^ ˃ 0.99 and Adj-R^2^ ˃ 0.98 for both analytes signify a high agreement between the obtained laboratory data and the model-predicted data. Adeq Precision (AP), as an indicator of the signal-to-noise ratio, is desirable in ratios > 4. In this research, the AP indexes were 65.66 and 44.32 for JG and SO, respectively, confirming the model’s high ability to predict the results.

**Table 3:** The results of ANOVA.

Source	DF	JG				SO			
		Sum of squares	Mean square	F-value	*P*-value	Sum of squares	Mean square	F-value	*P*-value
Model	14	12,180.43	870.03	360.24	< 0.0001	8304.85	593.20	188.24	< 0.0001
A	1	2613.97	2613.97	1082.33	< 0.0001	1012.44	1012.44	321.27	< 0.0001
B	1	1504.64	1504.64	623.01	< 0.0001	1713.32	1713.32	543.67	< 0.0001
C	1	2036.70	2036.70	843.31	< 0.0001	1776.07	1776.07	563.59	< 0.0001
D	1	1738.25	1738.25	719.74	< 0.0001	437.59	437.59	138.86	< 0.0001
AB	1	0.9653	0.9653	0.3997	0.5368	23.43	23.43	7.43	0.0156
AC	1	7.04	7.04	2.91	0.1085	2.96	2.96	0.9388	0.3480
AD	1	1.66	1.66	0.6864	0.4204	3.65	3.65	1.16	0.2990
BC	1	8.66	8.66	3.59	0.0778	1.90	1.90	0.6043	0.4490
BD	1	32.75	32.75	13.56	0.0022	9.00	9.00	2.86	0.1117
CD	1	14.88	14.88	6.16	0.0254	5.02	5.02	1.59	0.2263
A^2^	1	1602.52	1602.52	663.54	< 0.0001	1274.83	1274.83	404.53	< 0.0001
B^2^	1	2829.18	2829.18	1171.44	< 0.0001	1631.00	1631.00	517.55	< 0.0001
C^2^	1	501.25	501.25	207.55	< 0.0001	932.67	932.67	295.96	< 0.0001
D^2^	1	707.11	707.11	292.78	< 0.0001	850.97	850.97	270.03	< 0.0001
Residual	15	36.23	2.42			47.27	3.15		
Lack of fit	10	18.04	1.80	0.4957	0.8386	29.22	2.92	0.8091	0.6732
Pure error	5	18.19	3.64			18.05	3.61		
Cor total	29	12,216.65				8352.12			
Model summary statistics									
Precision		JG					SO		
		R^2^	R^2^-Adj	AP			R^2^	R^2^-Adj	AP
		0.9970	0.9943	65.66			0.9943	0.9891	44.32

Afterward, the assumption of data normality was examined. For this purpose, plots of actual values versus predicted values, normal distribution, and the distribution of residuals against the number of experiments were generated (Fig. S6). The plot of predicted values versus actual values is among the key tools for evaluating model performance in RSM. Ideally, all points should lie close to a straight 45-degree line, indicating good agreement between predicted and actual values. A large deviation of points from the straight line suggests reduced model accuracy or the presence of unpredictable factors and systematic errors. As shown in Figs. S6a and S6b, the points are well-distributed around the 45-degree line, demonstrating the model’s accurate prediction of actual values. The normal distribution plot of residuals is used to assess the normality of residuals. In this plot, standardized residuals are plotted against predicted values. The closeness of the points to the diagonal line indicates that the residuals follow a near-normal distribution, suggesting the model is well-fitted. According to Figs. S6c and S6d, the points are closely aligned along the diagonal (normal distribution line), confirming the normality of residuals. The residual scatter plot against the number of experiments illustrates the residuals, thereby representing the differences between observed and predicted values (Figs. S6e and S6f). A random distribution of residuals around the zero axis indicates normality and the absence of any specific trend, reflecting the model’s adequacy. As shown in Figs. S6e and S6f, the residuals are randomly distributed around the zero axis, with no discernible trend.

In the present study, 3D surface plots were employed for each reaction to illustrate the interaction effects between pairs of variables involved in the removal process of JG and SO dyes. The curvatures observed in these plots may suggest the presence of interactions between variables that play a role in the adsorption process.

**Fig. 2:**
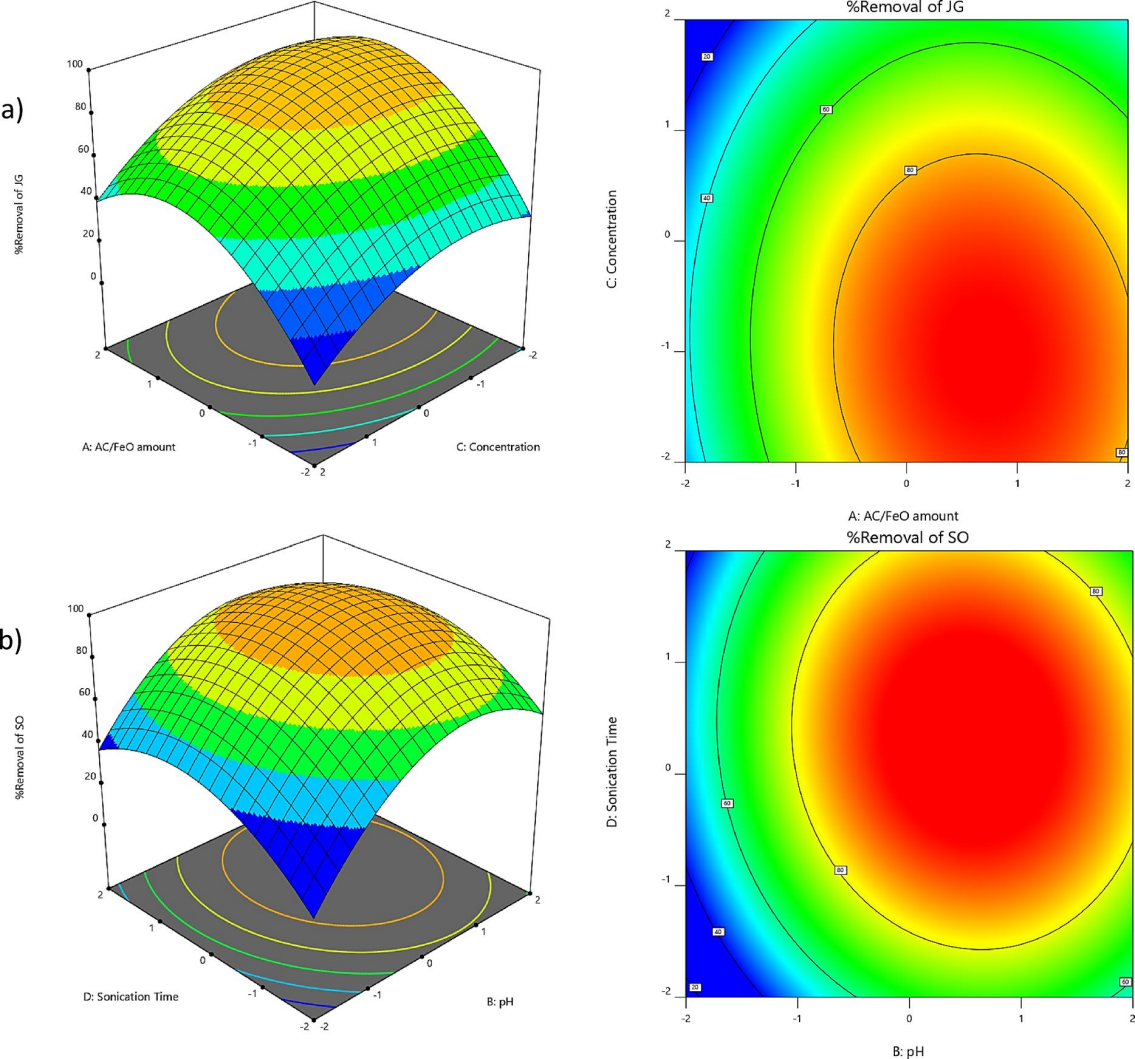
Three-dimensional plots of interactions for removal of dyes.

As shown in Fig. 2a, the dye removal percentage is expressed as a function of the AC/FeO nanocomposite dosage, representing that the removal efficiency increases with the increase in nanocomposite dosage. In other words, as the adsorbent dosage increases, the contact surface and active binding sites are enhanced. Consequently, the presence of more binding sites provides more effective functional groups, which play a significant role in JG dye removal. Thus, as depicted in Fig. 2a, increasing the adsorbent dosage facilitates the formation of more bonds between JG cations and the functional groups of the adsorbent. This increase in interactions between JG molecules and the adsorbent surface leads to enhanced adsorption from the aqueous solution, subsequently improving removal efficiency. These results are consistent with the findings of Khawaja et al. (2021), who studied the removal of malachite green using graphene oxide decorated with cellulose and copper nanoparticles as the adsorbent^49^.

Figure 2a illustrates the variations in the dye removal process as influenced by changes in JG concentration and AC/FeO nanocomposite dosage. The results show that reducing the JG concentration and increasing the nanocomposite dosage enhance dye removal efficiency. These changes follow a smooth trend over the reaction time. Besides, since the active surface area of a given adsorbent is fixed, increasing the JG concentration leads to a decline in removal efficiency due to the limited availability of active sites on the adsorbent surface. In essence, adsorbents possess a finite number of adsorption sites, and their efficiency diminishes when these sites become saturated. As the dye concentration continues to increase, the disturbance in JG adsorption onto the adsorbent surface intensifies, further reducing removal efficiency. These findings align with those of Yang et al. (2022), who investigated the removal of enrofloxacin (ENF) and Rhodamine B (RhB) using graphene oxide (GO)^50^. Similarly, Asfaram et al. (2015) reported comparable results in their study on the removal of Auramine-O using ZnS:Cu nanoparticles loaded on AC (ZnS:Cu-NP-AC) as an adsorbent^51^.

The SO removal process was completed within 25 min, with samples collected at intervals of 5, 10, 15, 20, and 25 min to examine the changes in adsorption over sonication time. Figure 2b depicts this trend under conditions of pH 7 and a nanocomposite dosage of 0.023 g. As can be seen, the highest adsorption occurred within the first 10 min, after which the adsorption rate slowed and reached equilibrium at around 18 min. According to the data presented in Fig. 2b, the maximum adsorption of JG and SO dyes was achieved during short sonication periods, highlighting the critical role of ultrasound in the mass transfer process. Similarly, Masoudinia et al. (2024) studied the removal of cephalexin (CFX) and eosin B (EB) using a magnetic chitosan/zinc oxide nanocomposite (CS/ZnO-Fe_2_O_4_) and reported comparable results. The rapid adsorption was attributed to the nanoscale size and high surface area of the adsorbent^52^.

One of the key characteristics of the AC/FeO nanocomposite is its point of zero charge (pH_pzc_), which plays a crucial role in pollutant removal. In this research, the pH_pzc_ of AC/FeO nanocomposite was determined to be 5.3, indicating that the nanoparticle surface carries a positive charge at pH < 5.3 and a negative charge at pH > 5.3. Therefore, the interaction between the nanoparticle surface and pollutant species is highly dependent on the pH of the environment. As shown in Fig. 2b, in acidic conditions (pH < 5.3), the AC/FeO nanocomposite exhibits low efficiency in SO dye removal. In contrast, SO dye removal significantly increased in alkaline conditions (pH > 5.3). This behavior is attributed to electrostatic interactions between the surface charge of the nanoparticles and the dye in the solution. Based on the results, the highest SO removal efficiency was observed at pH 7. Similarly, Phuong and Loc (2022) reported an optimal pH of 7 for SO removal^53^.

### Optimization of variables

In the 3D plots, the optimal conditions for independent variables were illustrated in pairs; however, these plots alone are insufficient to determine the overall optimal conditions. Therefore, parameter optimization was conducted to identify conditions under which the maximum removal efficiency could be achieved while ensuring all variables were at their optimal levels. Table 4 presents the values of each independent variable at the identified optimal points, along with the predicted and experimental results. The results show that the highest removal efficiency using the AC/FeO magnetic nanocomposite was 95.79% and 97.60% for JG and SO dyes, respectively, under optimal conditions. These conditions are a concentration of 20 mg L^−1^, sonication time of 18 min, nanocomposite dosage of 0.023 g, and pH of 7. The minimal difference between the predicted and experimental results indicates that CCD can be employed to evaluate the removal process of JG and SO dyes effectively using the AC/FeO magnetic nanocomposite and accurately identify the optimal pH.

**Table 4:** Optimal conditions of DOE.

Optimal conditions				%Removal ± RSD (n = 3)			
				JG		SO	
AC/FeO amount	pH	Concentration	Sonication time	Predicted	Experimental	Predicted	Experimental
0.023 g	7	20 mg L^−1^	18 min	94.18	95.79 ± 2.38	96.82	97.60 ± 1.95

### Recovery of adsorbent

**Fig. 3:**
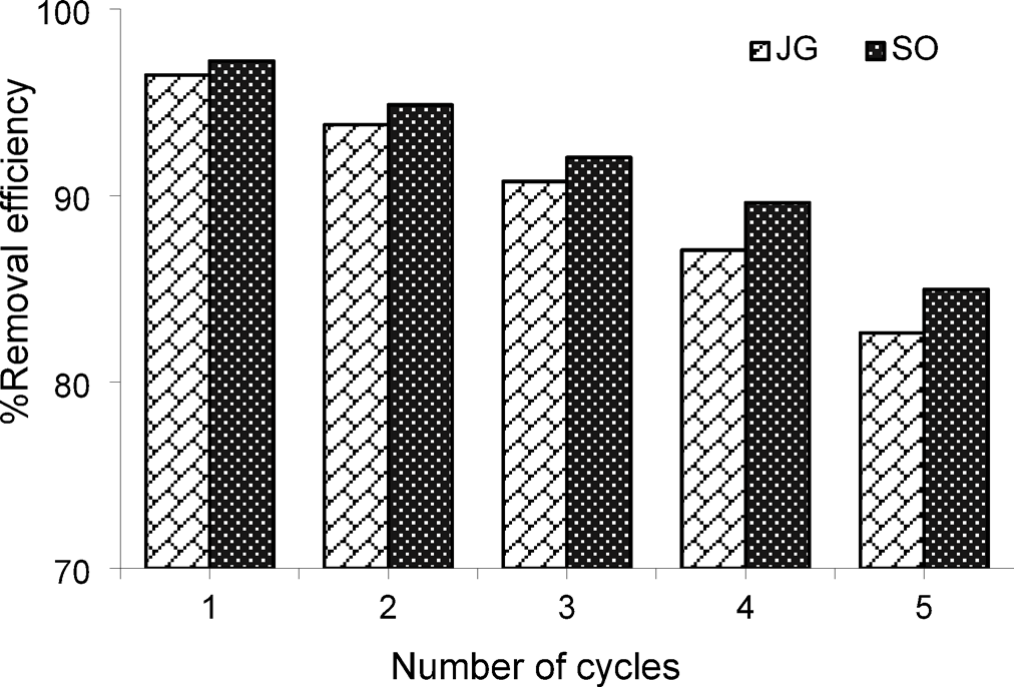
Efficiency of AC/FeO nanocomposite for dyes removal in the cycles.

The reusability of the AC/FeO magnetic nanocomposite for removing JG and SO dyes from aqueous solutions is a critical and practical topic in water and wastewater treatment. Repeated use of adsorbents provides significant economic benefits and helps reduce solid waste and the associated environmental impacts of disposing of spent adsorbents. The adsorption efficiency after each recovery cycle must be assessed to evaluate the recyclability of the adsorbent. Accordingly, five regeneration tests were conducted for the AC/FeO magnetic nanocomposite under optimal conditions and subsequent desorption. As shown in Fig. 3, the removal efficiency decreased slightly after each cycle, even with full recovery. Nevertheless, the performance of the AC/FeO magnetic nanocomposite demonstrated that this adsorbent remains effective for reuse even after five consecutive cycles.

### Real samples analysis

We explored the application of the method in removing JG and SO dyes from various water sources, including tap water, river water, and wastewater. The effectiveness of the proposed approach was evaluated by performing the adsorption process on the samples in three replicates. Subsequently, the average removal rates of JG and SO in these water samples were calculated. As can be seen (Table S1), the removal efficiencies for JG and SO dyes in real water samples ranged from 87.65 to 94.81%. These results suggest that the use of the AC/FeO magnetic nanocomposite for removing JG and SO dyes from diverse water matrices, including simulated real-world conditions, highlights its potential for application in complex environments.

## Conclusion

The present study synthesized the AC/FeO magnetic nanocomposite for the adsorption and removal of JG and SO dyes. The synthesized nanocomposite was characterized using VSM, XRD, BET, FT-IR, and SEM analyses. The pH_pzc_ for the AC/FeO magnetic nanocomposite was determined to be 5.3. The AC/FeO nanocomposite has a surface area of 329.56 m^2^ g^−1^, a pore size of 3.67 nm, and a pore volume of 0.289 cm^3^ g^−1^. RSM based on CCD was employed to investigate the adsorbent’s behavior in removing JG and SO dyes and to determine the optimal adsorption conditions. The results revealed that the optimal conditions for the removal of JG and SO dyes using the AC/FeO magnetic nanocomposite were a sonication time of 18 min, a dye concentration of 20 mg L^−1^, a nanocomposite dosage of 0.023 g, and a pH of 7. Under these conditions, the removal efficiencies were 95.79% and 97.60% for JG and SO, respectively. Overall, this study demonstrated that RSM is a reliable tool for optimizing the conditions for JG and SO dye removal using the AC/FeO adsorbent. The adsorbent’s efficiency under optimal conditions declined by approximately 20% after five consecutive cycles. This study underlines the potential of the AC/FeO magnetic nanocomposite as a promising and efficient adsorbent for removing JG and SO dyes from aqueous samples. Overall, the findings indicate that the AC/FeO magnetic nanocomposite can serve as an effective and economical adsorbent for removing JG and SO dyes from aqueous solutions.

## Electronic supplementary material

Below is the link to the electronic supplementary material.

Supplementary Material 1

## Data Availability

All data generated or analyzed during this study are included in this published article.
