# Supplementary material for: Application of response surface methodology for modeling adsorption of janus green and safranin-O on magnetic nanocomposite from aqueous solutions
Source: Sci Rep. 2025 Aug 25;15:31252. doi: 10.1038/s41598-025-02644-1 (PMC12378200; doi:10.1038/s41598-025-02644-1)
Supplement: Supplementary file 1 — Supplementary Material 1 [file 41598_2025_2644_MOESM1_ESM.docx]

**Supplementary Material**

**Title**

**Application of response surface methodology for modeling adsorption of janus green and safranin-O on magnetic nanocomposite from aqueous solutions**

**Figures captions**

**Fig. S1.** FT-IR of AC/FeO nanocomposite.

**Fig. S2.** XRD of AC/FeO nanocomposite.

**Fig. S3.** VSM of AC/FeO nanocomposite.

**Fig. S4.** SEM of a) AC and b) AC/FeO nanocomposite.

**Fig. S5.** BET of AC/FeO nanocomposite.

**Fig. S6.** Plot of predicted values versus actual values for a) JG, b) SO; Normal plots of residuals for c) JG and d) SO; Plot of residuals versus run number for e) JG and f) SO.

**Tables captions**

**Table S1.** Results of real samples under the optimum conditions (n= 3).


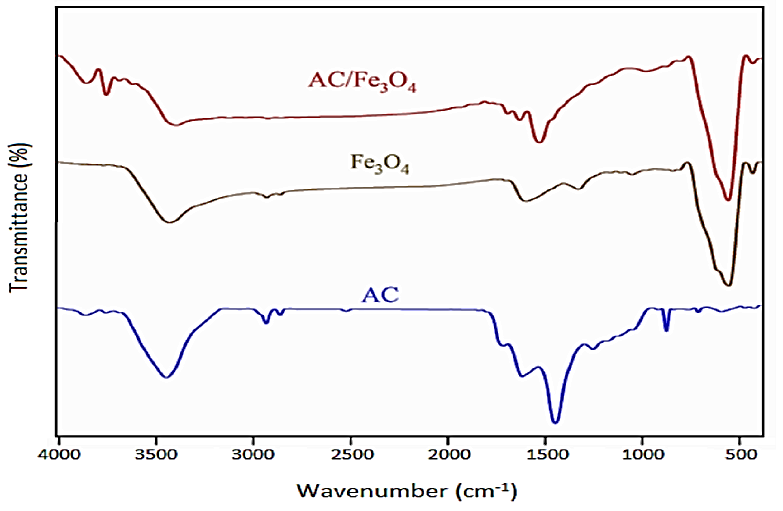


**Fig. S1.** FT-IR of AC/FeO nanocomposite.


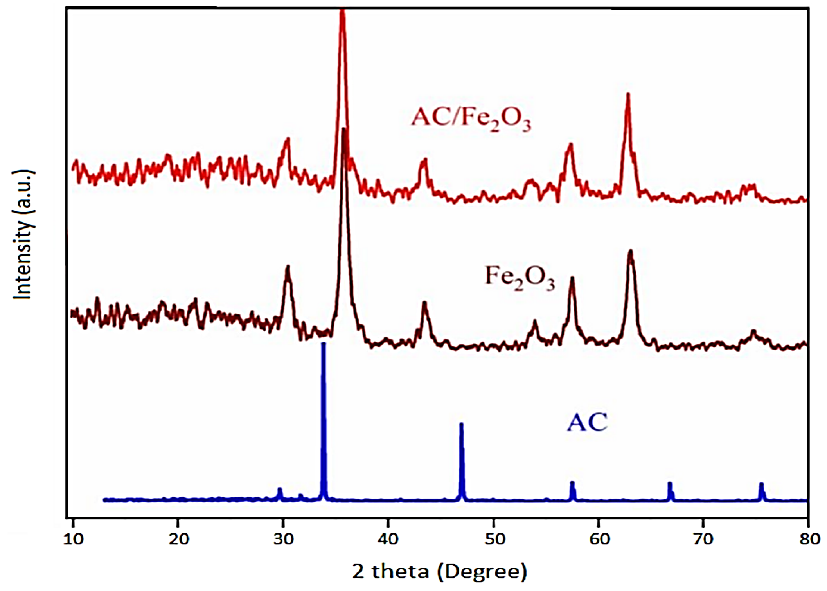


**Fig. S2.** XRD of AC/FeO nanocomposite.


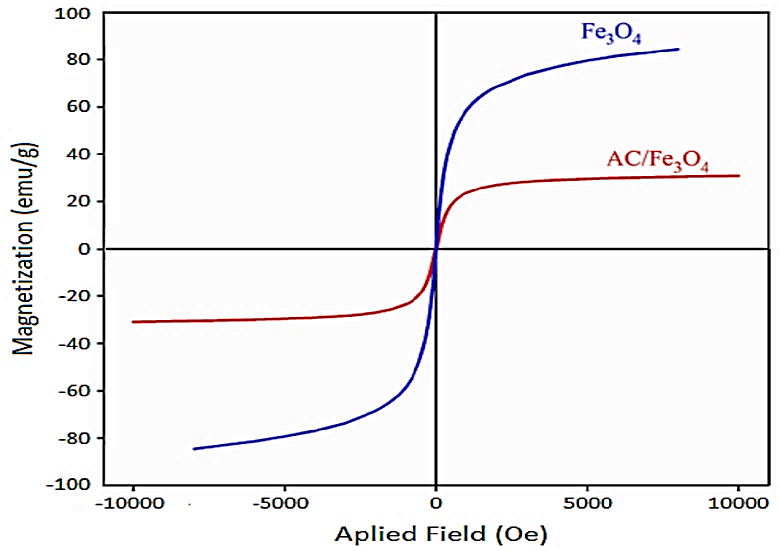


**Fig. S3.** VSM of AC/FeO nanocomposite.

| 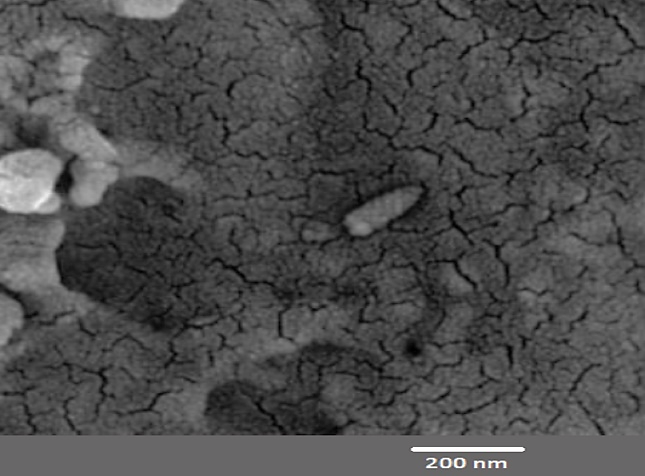  a) | 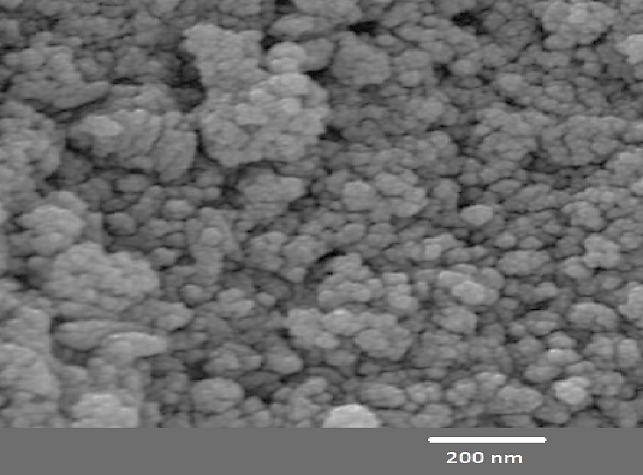  b) |
| --- | --- |
| **Fig. S4.** SEM of a) AC and b) AC/FeO nanocomposite. | |


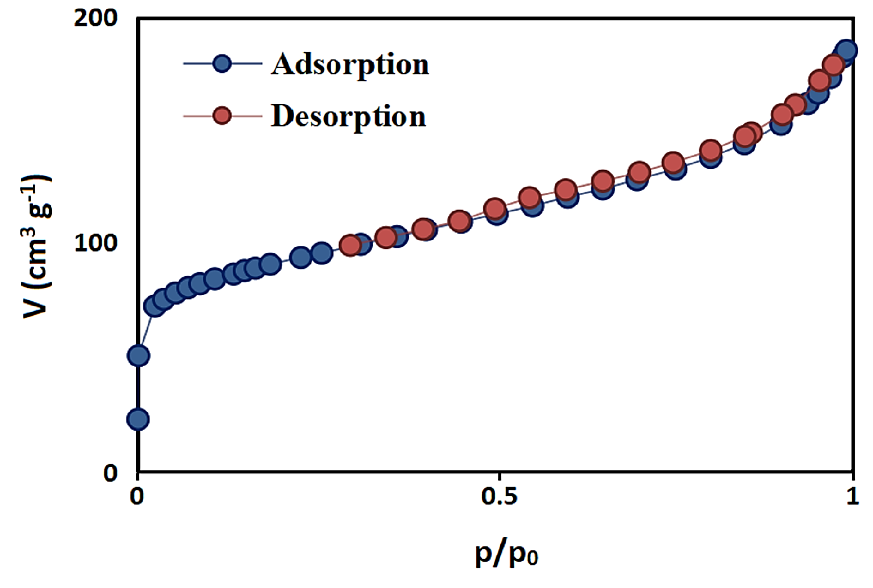


**Fig. S5.** BET of AC/FeO nanocomposite.

| 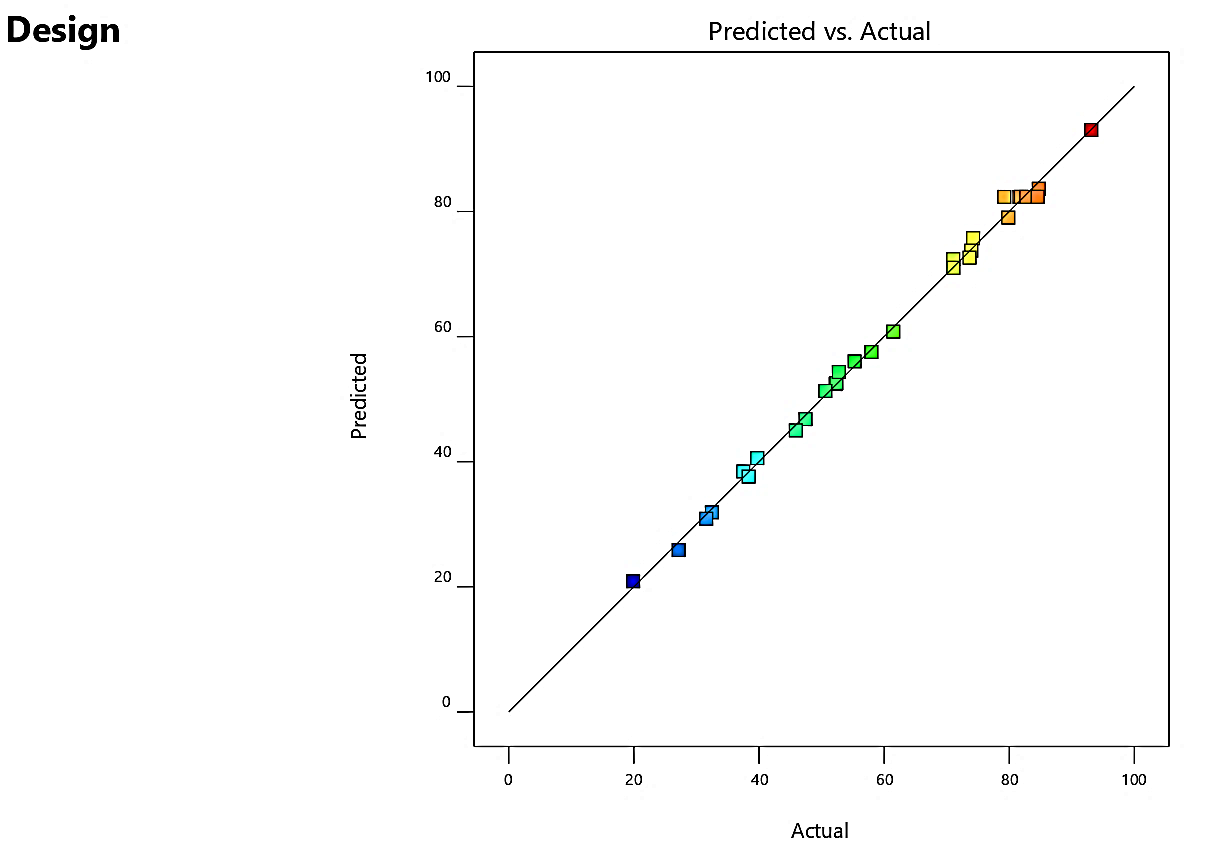  a) | 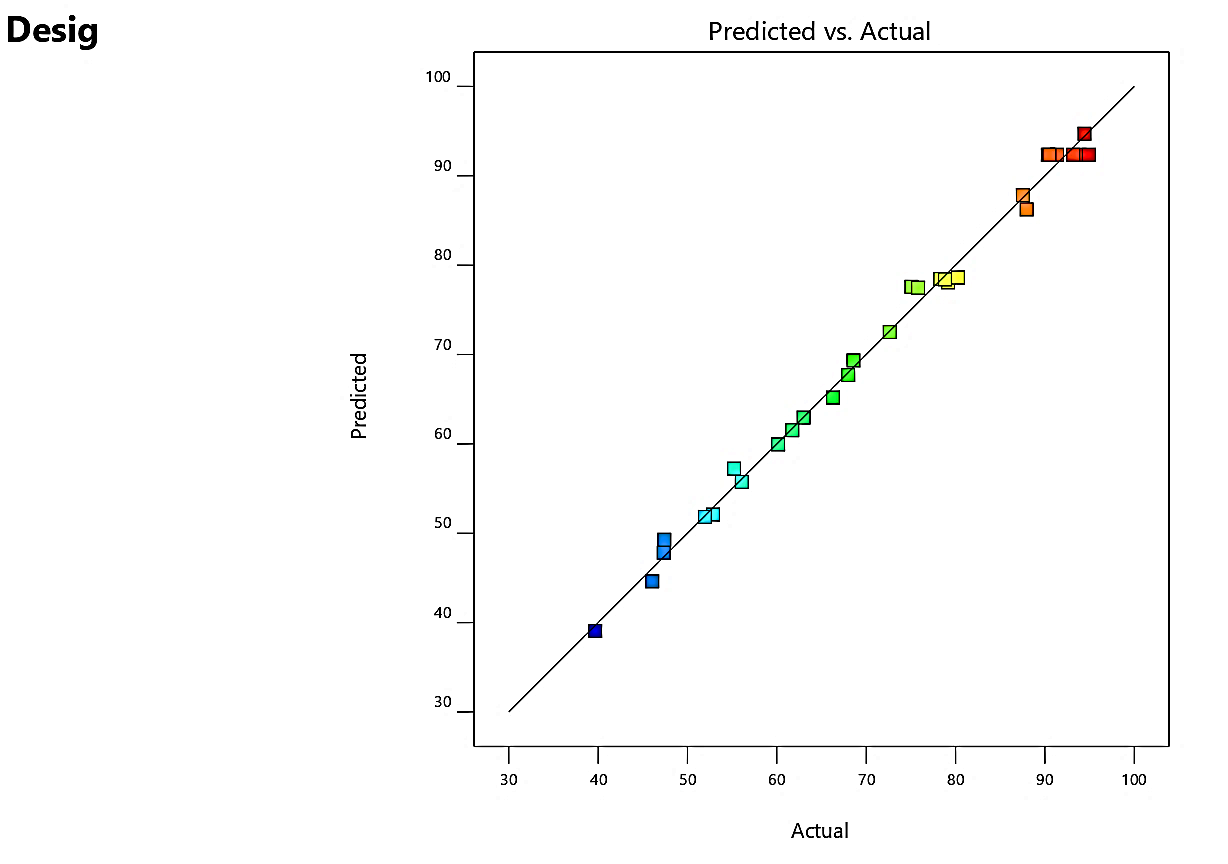  b) |
| --- | --- |
| 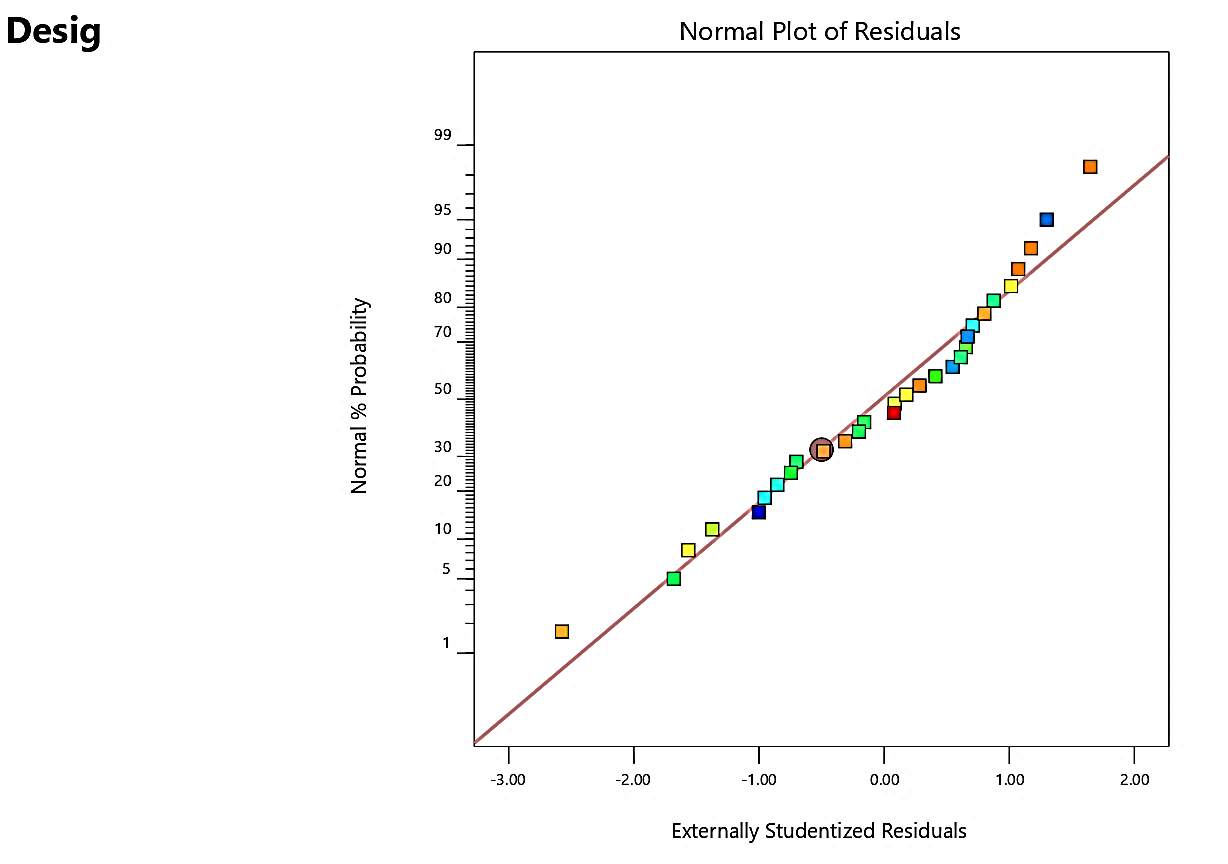  c) | 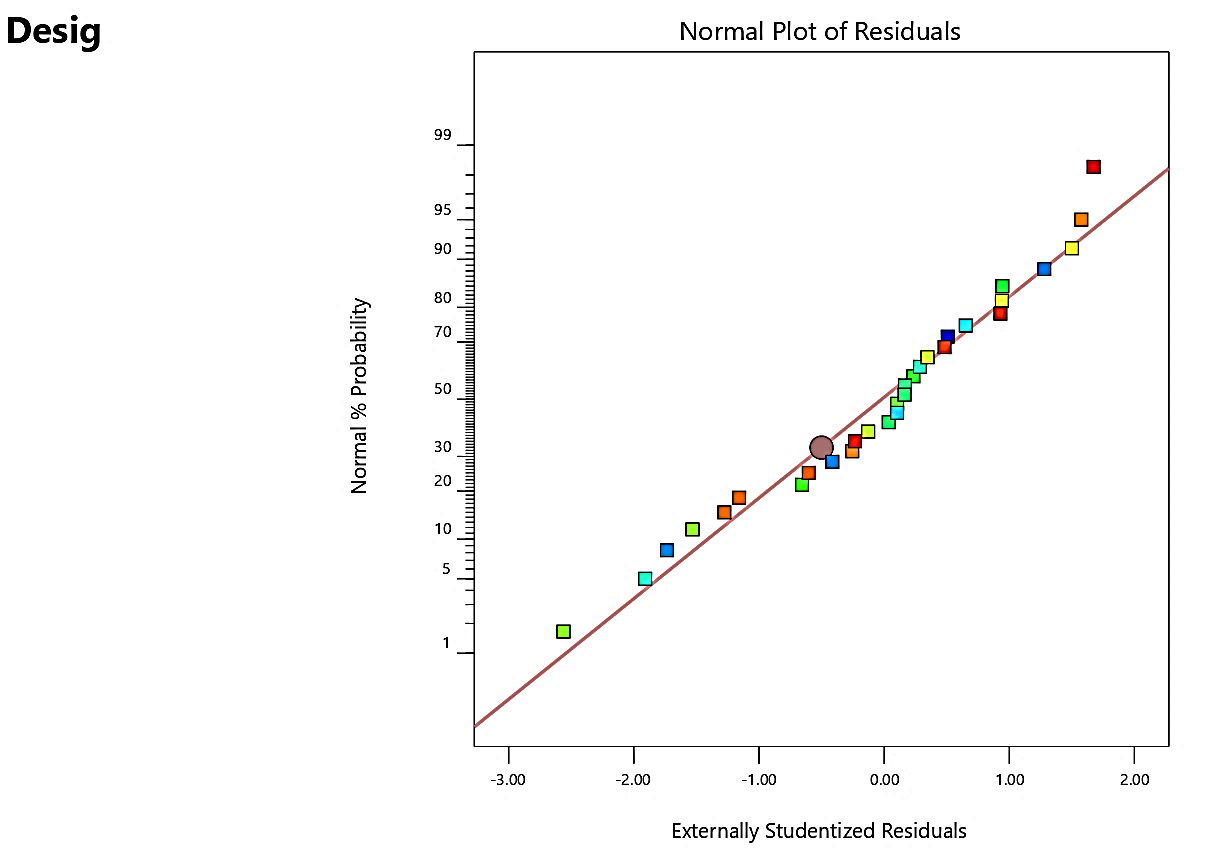  d) |
| 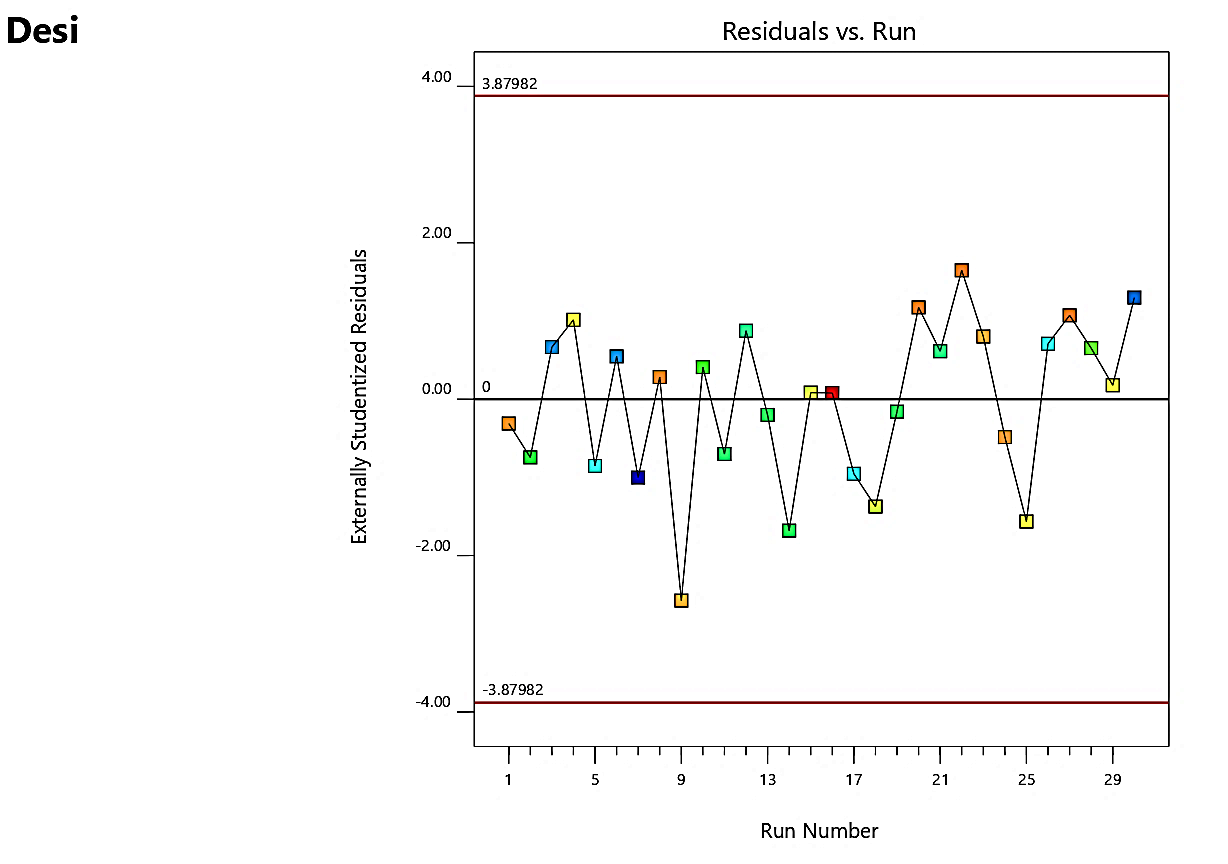  f) | 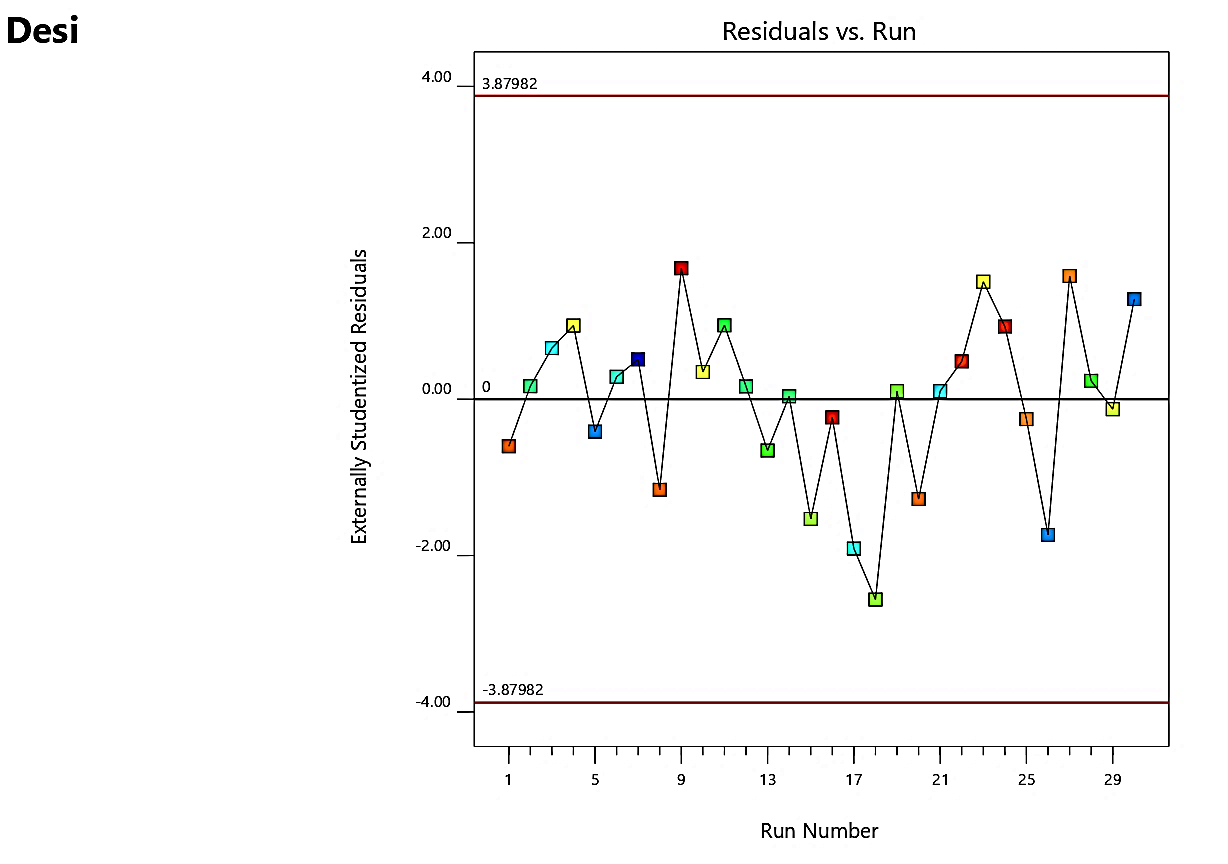 |
| **Fig. S6.** Plot of predicted values versus actual values for a) JG, b) SO; Normal plots of residuals for c) JG and d) SO; Plot of residuals versus run number for e) JG and f) SO. | |

e)

c)

a)

**Table S1.** Results of real samples under the optimum conditions (n= 3).

| Samples | %Removal ± %RSD | | |
| --- | --- | --- | --- |
|  | JG | SO | |
| Tap water | 94.39 ± 1.9 | | 94.81 ± 2.0 |
| River water | 91.94 ± 2.3 | | 93.94 ± 2.7 |
| Wastewater | 87.65 ± 2.4 | | 89.78 ± 2.5 |
